# Accelerated diversifications in three diverse families of morphologically complex lichen-forming fungi link to major historical events

**DOI:** 10.1038/s41598-019-44881-1

**Published:** 2019-06-28

**Authors:** Jen-Pan Huang, Ekaphan Kraichak, Steven D. Leavitt, Matthew P. Nelsen, H. Thorsten Lumbsch

**Affiliations:** 1 Integrative Research Center, The Field Museum, Chicago, IL 60605 USA; 2 Department of Botany, Faculty of Science, Kasetsart University, Bangkok, Thailand; 3 Department of Biology and M. L. Bean Life Science Museum, Brigham Young University, Provo, UT 84602 USA; 4 Biodiversity Research Center, Academia Sinica, Taipei, Taiwan

**Keywords:** Speciation, Phylogenetics

## Abstract

Historical mass extinction events had major impacts on biodiversity patterns. The most recent and intensively studied event is the Cretaceous – Paleogene (K-Pg) boundary (ca. 66 million years ago [MYA]). However, the factors that may have impacted diversification dynamics vary across lineages. We investigated the macroevolutionary dynamics with a specific focus on the impact of major historical events such as the K-Pg mass extinction event on two major subclasses – Lecanoromycetidae and Ostropomycetidae – of lichen-forming fungi and tested whether variation in the rate of diversification can be associated with the evolution of a specific trait state - macrolichen. Our results reveal accelerated diversification events in three families of morphologically complex lichen-forming fungi – Cladoniaceae, Parmeliaceae, and Peltigeraceae – which are from the subclass Lecanoromycetidae and mostly composed of macrolichens, those that form three dimensional structures. Our RTT plot result for the subclass Lecanoromycetidae also reveals accelerated diversification. Changes in diversification rates occurred around the transition between Mesozoic and Cenozoic eras and was likely related to the K-Pg mass extinction event. The phylogenetic positions for rate increases estimated based on marginal shift probability are, however, scattered from 100 to 40 MYA preventing us from making explicit inference. Although we reveal that the phenotypic state of macrolichens is associated with a higher diversification rate than microlichens, we also show that the evolution of macrolichens predated the K-Pg event. Furthermore, the association between macrolichens and increased diversification is not universal and can be explained, in part, by phylogenetic relatedness. By investigating the macroevolutionary dynamics of lichen-forming fungi our study provides a new empirical system suitable to test the effect of major historical event on shaping biodiversity patterns and to investigate why changes in biodiversity patterns are not in concordance across clades. Our results imply that multiple historical events during the transition from Mesozoic to Cenozoic eras, including the K-Pg mass extinction event, impacted the evolutionary dynamics in lichen-forming fungi. However, future studies focusing on individual lichen-forming fungal families are required to ascertain whether diversification rates are associated with growth form and certain geological events.

## Introduction

The five mass extinctions in earth’s history had major impacts on biodiversity, reshaping entire ecosystems and resulting in dramatic changes in the diversity of major clades^1–4^. The latest of these biotic crises, the Cretaceous – Paleogene (K-Pg) boundary 66 million years ago (MYA)^5^, is well known due to the extinction of non-avian dinosaurs^6,7^. There is a growing body of evidence that the composition of contemporary biodiversity has been significantly shaped by this last mass extinction event. The loss of entire clades during the K-Pg boundary created new ecological opportunities facilitating rapid diversification of surviving lineages^2^. For example, new ecological opportunities might be related to the extinction of predators and competitors, new open niches that were previously occupied or newly formed because of new environmental conditions, and the evolution of novel traits – biological (e.g., new phenotype) and ecological (e.g., symbiosis). Supporting evidence includes fossil data demonstrating mass extinction of all non-avian dinosaurs, but also mass extinction in other clades of terrestrial life, such as crocodyliforms, snakes and lizards, birds, mammals^8–11^. In addition, recent comparative phylogenetic studies have indicated that the K-Pg mass extinction event may have facilitated the subsequent rapid diversifications in modern day birds, their lice, and frogs^12–14^. These specific shifts have been proposed to facilitate the radiation of major clades of mammals, such as rodents^15^, which account for approximately 42% of extant mammalian diversity^16^, and of plant clades with duplicated genomes^17^.

The impact of the K-Pg boundary on diversification of organisms with scant fossil records, such as fungi and some photosynthetic organisms, is less well known. An increase of fungal spores in the fossil record following the mass extinction event and deforestation indicates increased frequency of decomposing fungal species^18,19^. In contrast, photosynthetic organisms, such as plants, are hypothesized to have undergone mass extinction events after the K-Pg boundary due to severe global climatic change^20^. However, the impact of this mass extinction on symbiotic fungi, such as lichen-forming fungi with poor fossil record^21,22^ has not yet been studied. Lichens are symbiotic systems containing at least a fungal host and a mutualistic algal/cyanobacterial partner, although lichen symbioses can also involve bacteria, accessory algae, and endolichenic fungi. Lichens are unique biological systems because the macroevolutionary dynamics of the free-living relative to the two main partners of the system – photosynthetic land plants and fungi – have been hypothesized to react differently to the K-Pg event^18–20^. It is of evolutionary interest to understand whether the long-term evolutionary dynamics is determined by the hosts that construct and fill specific ecological niches or the obligate symbionts that determine the survival of the hosts.

**Figure 1:**
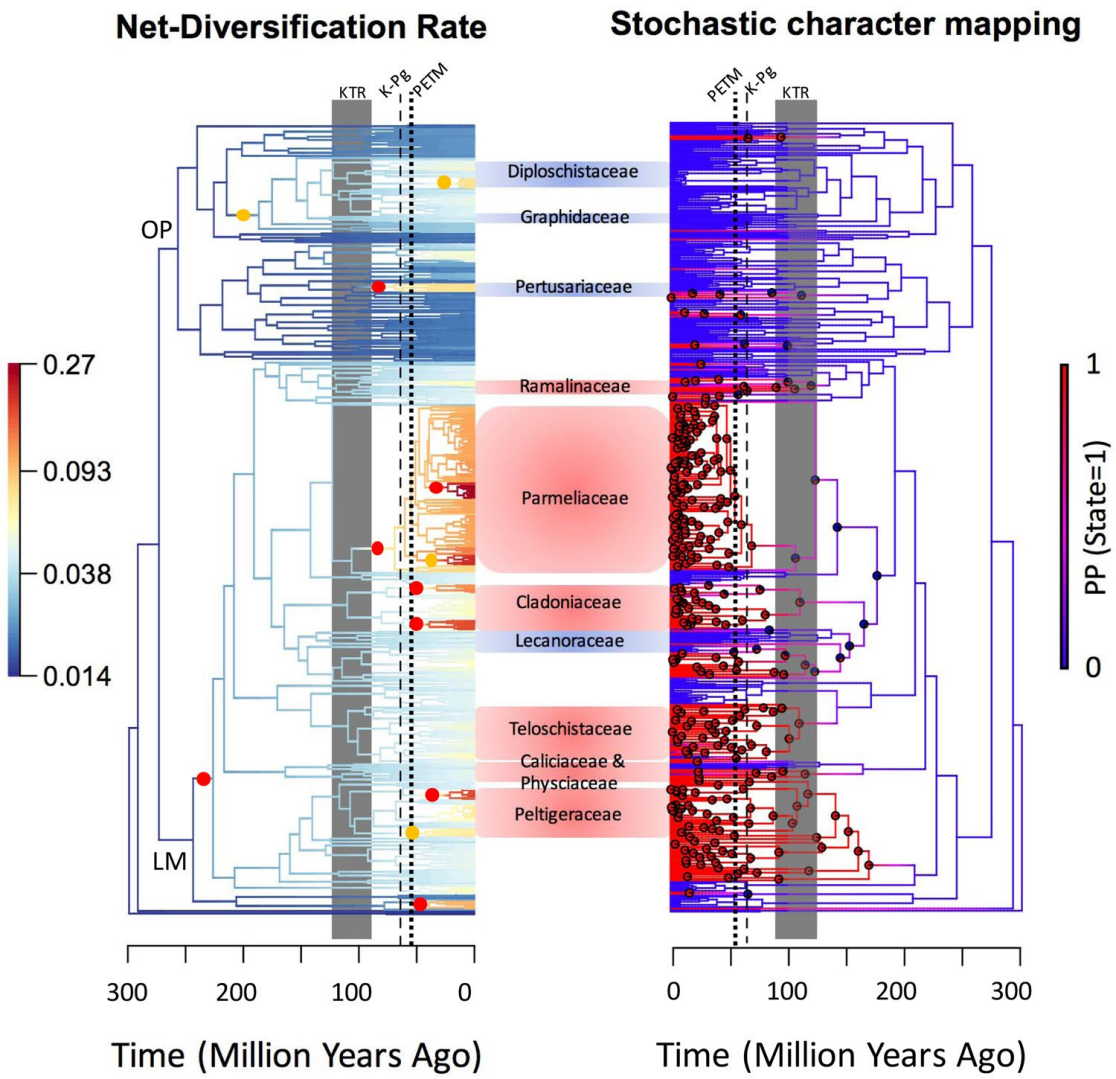
Changes in diversification dynamics through time (left) and the evolutionary history of microlichen versus macrolichen (right). Branch color of the left figure depicts the net-diversification rate estimated from a BAMM analysis with a warmer color indicating higher rates. Color dots in the left panel indicate the positions where diversification rate has shifted (red dots indicate a marginal shift probability >0.5 and yellow dots >0.3). Branch color of the right figure depicts the probability of being state 1 (macrolichen), where a warmer color indicates a higher probability. Pie charts on nodes indicate the estimate trait states, where only nodes with >10% estimated probability of being macrolichens are shown. Dash lines in both figures indicate the K-Pg boundary (66 MYA) and the Paleocene-Eocene thermal maximum (PETM); the grey shaded areas indicate the period of rapid angiosperm diversification (KTR). LM = Lecanoromycetidae, OP = Ostropomycetidae.

**Figure 2:**
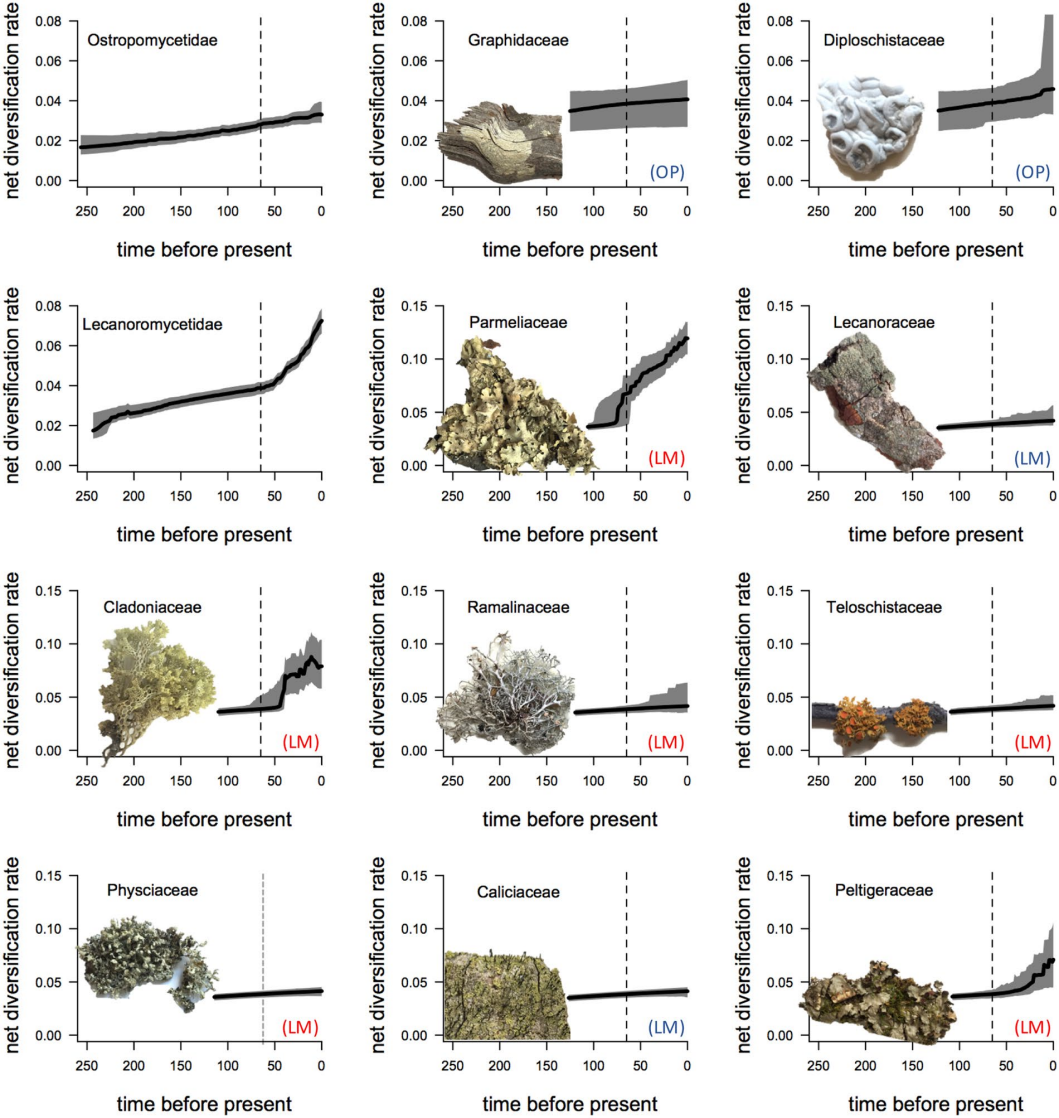
Net-diversification rate through time plots of the two subclasses and families with more than 500 species in these two main subclasses of lichen-forming fungi. Solid lines are the mean net-diversification rates and the grey areas depict the 95% probability densities. Vertical dash lines indicate 66 MYA. LM = Lecanoromycetidae, OP = Ostropomycetidae. Red color = lineages that are mostly macrolichens, blue color = microlichen lineages.

**Figure 3:**
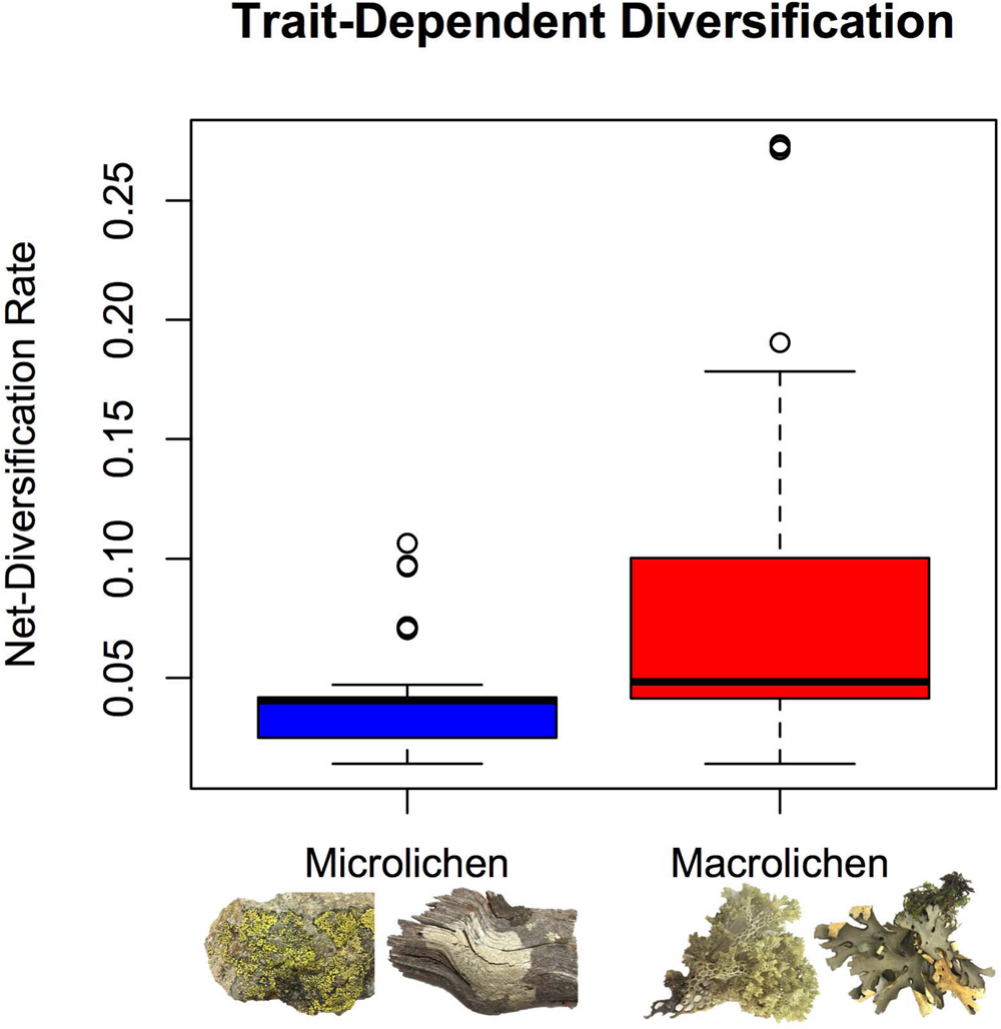
A boxplot for the estimated net-diversification rates for current microlichen (blue) and macrolichen (red) species.

Investigating the interplay of historical events on macroevolutionary dynamics can provide further important insight into the unevenly distribution of species richness across the tree of life. The uneven distribution of species richness among clades across the tree of life can be explained by multiple factors leading to different rates of extinction and diversification. Increased rates of diversification in a clade are often explained by adaptive traits that evolve in a clade, so called key innovations^23,24^. For example, it has been demonstrated that speciation rate in color-polymorphic birds is significantly higher than that in other monomorphic bird lineages^25^. While attributing a single trait to the radiation of a lineage can be overly simplistic, identifying these putative key innovations, in tandem with other factors, can provide a better insight into overall diversification processes^26^. Lichen-forming fungi are unusual among fungi in that they form long-lived vegetative structures (thalli) to house the photosynthetic partners and these thalli can have different growth forms to resemble crusts (microlichens) or be more complex (macrolichens) being either leaf-like (foliose) or shrubby (fruticose)^27^ (Figs 1–3). A number of recent studies addressed the identification of key innovations in various groups of organisms, including in lichen-forming fungi^28–38^. The evolution of macrolichens may represent an innovation, because it allows lichens to explore additional niche spaces and to develop different structures that are not constrained to the surface of substrates. The impact of evolving a macrolichen phenotype is however unequivocal based on empirical data. For example, it has been shown that clades with macrolichens are more species-rich than clades forming primarily microlichens in some families of lichen-forming fungi, but also some clades containing only microlichens have been found to be hyperdiverse^39–41^.

Here we examine how major historical events and different diversification rates associated with a trait have shaped the diversity of lichen-forming fungi. Current evidence suggests that the lichenized life-style evolved in fungi during the Devonian^22^. Devonian lichen fossils already exhibit very complex structure, and there are other earlier fossils that might represent simplified/early-stages of lichenized life style. Hence, lichen-forming fungal clades have persisted through major historical events and are also species-rich and phenotypically diverse^42^. Given the impact of the K-Pg mass extinction event on terrestrial life, particularly on photosynthetic organisms^20^, it is plausible to assume that diversity of lichens, a mutualistic system of fungi and photosynthetic partners^27^, was also negatively affected by this event. On the other hand, should the K-Pg mass extinction event lead to extinction of epiphytic plant competitors, lichens that have similar morphology and ecological preference might have been benefited from the event and diversified to fill the newly open niches. Within a comparative phylogenetic context, we studied the association between the evolution of different trait states and major historical events and tested whether this putative evolutionary innovation may have accelerated diversification in lichen-forming fungi. Specifically, we investigated the macroevolutionary dynamics in the two major subclasses of lichen-forming fungi, Lecanoromycetidae and Ostropomycetidae, representing >90% of the species diversity in the class Lecanoromycetes (14900 estimated species). We used a recently reconstructed molecular phylogeny and classification of the two subclasses^43^ and a recently published monograph of the phylum Ascomycota that comprehensively reviewed and estimated the number of species per genus^42^. Our results provide a diversification history of lichen-forming fungi in general and also provide specific novel insights into how major historical events, such as the K-Pg mass extinction, might (or might not) impact diversification of mutualistic fungi.

## Results and Discussion

Our current understanding of how historical events and evolutionary innovations might have affected variation in biodiversity patterns across geological periods and evolutionary lineages is largely derived from only a few well studied organismal groups, which can be incomplete and biased. Although the study of macroevolutionary dynamics has been revolutionized by molecular phylogenetic and comparative methods, these methods assume that the studies have complete taxon sampling or have been adjusted for missing samples. Therefore, studies have been restricted to vertebrates or some plant and insect groups that the species richness and the extant biodiversity pattern are better understood. Here, we extend our knowledge about the variation in diversification histories and patterns across different evolutionary lineages by studying two major clades of lichen-forming fungi. Our analysis of the macroevolutionary dynamics across lichen-forming fungi reveals multiple accelerated diversification events between 100 and 40 MYA, which encompasses the K-Pg boundary in several clades (based on estimated marginal shift probabilities; Fig. 1). Although most shifts in diversification rate occurred after the K-Pg boundary, some diversification shifts were also found to have occurred prior to the K-Pg boundary (Figs 1 and 2). Interestingly, our results suggest that no lineage of lichen-forming fungi within Lecanoromycetes experienced decelerated diversification. Accelerated diversification rates are particularly present in clades including macrolichens, and notably absent from most clades comprised microlichens (Figs 1 and 2). Significantly higher net-diversification rates are pronounced in clades consisting predominantly of macrolichens (Fig. 3). However, the hypothesis that such association can be explained by phylogenetic relatedness instead of the trait states *per se* cannot be ruled out (accessed with STRAPP^44^, *P*s = 0.223, 0.187, and 0.222 using Spearman’s rho, Pearson correlation, and Mann-Whitney test, respectively). We note that in comparison to macrolichens, microlichen genera have not been subject to intensive molecular-based species delimitation and taxonomic studies, hence it cannot be ruled out that species diversity in microlichens might be underestimated. Future studies are needed to evaluate whether our reported pattern is an artifact due to inconsistent taxonomic efforts across lineages. We discuss how our finding sheds new lights on the effects of major historical events, ecological opportunities, and evolutionary innovations on biological diversification in the following sections.

Our finding implies that historical mass extinction events, and specifically the K-Pg event, might have impacted the diversification of lichen-forming fungi, as have been shown in other organismal groups^1,2^. However, in contrast to the predicted pattern of a decline in species richness that has been hypothesized for photosynthetic organisms^20^, the K-Pg extinction appears to have had positive effect on diversification in lichen-forming fungi. There are multiple instances of shifts in diversification rate after the K-Pg boundary, especially in the subclass Lecanoromycetidae (Figs 1 and 2). Furthermore, there is a sudden increase in diversification after the K-Pg boundary based on the estimated diversification rate through time in Lecanoromycetidae, whereas the estimated diversification rate increases gradually and continuously in subclass Ostropomycetidae (Fig. 2).

It is worth noting that many lichen-forming fungi can utilize different photosynthetic symbionts (both algae and cyanobacteria), and switches between different algal partners have been reported in empirical studies^22,27,28^. Therefore, mass extinction of other competitive evolutionary lineages such as other land plants might have led to the subsequent rapid diversification of the surviving lichen-forming fungal lineages that could develop new mutualistic relationships with the remaining algal/cyanobacterial partners. Nevertheless, given the uncertainty in phylogenetic dating and the geological time scale focused in our study, there were other major historical events and hypotheses that may also explain the observed pattern. For example, prior to the K-Pg event, the Cretaceous Terrestrial Revolution (KTR; from 125 to 80 MYA; Fig. 1) where angiosperms diversified rapidly could also account for the accelerated diversification in the epiphytic lichens^45,46^. The increase in diversification rate found in certain lichen-forming fungal lineages is not necessarily a result of mass extinction event of other lineages resulting in open niche space. The diversification and multiplication of angiosperms lineages might have provided new niches for epiphytic lichen radiation, e.g. novel durable and diverse substrates on vascular plants. Our study only provides a general time frame of lichen diversification and future studies are needed to specifically test the effect of different historical events and their interactions on diversification rate of lichen-forming fungi.

Our results imply that the evolution of macrolichens was an evolutionary innovation that helped certain lineages to rapidly diversify when new ecological opportunity emerged. Specifically, macrolichens might have diversified because of the new niches created by rapidly diversifying angiosperms during KTR; furthermore, those lichen-forming fungal lineages that survived the K-Pg mass extinction event might have rapidly filled the open niches resulted from the extinction of competitors. The three families showing patterns of acceleration in diversification rates based on the rate through time plots are from the subclass Lecanoromycetidae and form predominately macrolichens (Fig. 2). Such inference is further supported by the fact that the estimated net-diversification rate is significantly higher for macrolichens than microlichens (*P* < 0.001 based on *t*-test; Fig. 3).

It is tempting to infer that the evolution of macrolichens, which may serve ecological functions like epiphytes, might prosper due to the extinction of competitors after the K-Pg boundary or because of the angiosperm radiation during KTR. It is, however, more difficult to determine how and why the macrolichen phenotype facilitated accelerated diversification rates. There are also patterns of accelerated diversification rate found in the Ostropomycetidae subclass, which consists of mainly microlichens (e.g., Pertusariaceae; Fig. 1). Some families predominately forming macrolichens in subclass Lecanoromycetidae did not undergo diversification rate increase. Furthermore, such association between macrolichen and a higher diversification rate disappeared when the phylogenetic relatedness among taxa were taken into consideration (STRAPP result). Note that, to test for trait-dependent hypothesis using STRAPP, it often requires a phylogeny with thousands of tips and a dozen of independent origins of the putative adaptive phenotype^47^. Most empirical data sets likely do not have the statistical power to detect trait-dependent diversification pattern using STRAPP even if the pattern exists. Additional comparative phylogenetic studies that focus on individual families, thus controlling for the phylogenetic lineage specific effect, will be required to explicitly test whether evolving the macrolichen growth form is an evolutionary innovation.

Macrolichens belong to young lineages nested within larger microlichen clades, and arose independently multiple times from microlichens at various points of time based on ancestral state reconstruction (Fig. 1). Specifically, both symmetric and asymmetric models of discrete character evolution fit the trait data and the phylogeny well (transition rate between states is 0.00136 for symmetric model with a log likelihood value of −132.8879; the transition rates are 0.00107 and 0.00149 from macrolichen to microlichen and *vice versa*, respectively with a log likelihood value of −132.6705; there is no statistical significance between the two models given the data, *P* = 0.49), so the result from the symmetric model is shown in Fig. 1. The early divergences occurred between microlichens, and it was not until ca. 200 MYA that macrolichens started to evolve from microlichens. Note that, macrolichens form a composite trait state that can be further divided into finer categories as growth forms – e.g., foliose and fruticose – and a diversity of different structure variations also exist within the growth forms^27^. It is likely that the different growth forms may represent different lineage-specific evolutionary innovations. This evolutionary innovation predated the evolutionary diversification of angiosperms and the K-Pg boundary in Lecanoromycetidae, but was synchronized with the evolutionary diversification of angiosperms in Ostropomycetidae (Fig. 1). Our result corroborates the finding from a previous study focusing on the evolutionary diversification among major Ascomycota lineages^48^. There is, however, substantial variation in the evolutionary history of microlichens versus macrolichens across lineages. As it predated both KTR event and the K-Pg boundary, the evolution of macrolichens in certain lineages may be pre-adaptive and led to subsequent accelerated diversification once provided with new ecological opportunities created by diversifying angiosperms during KTR and/or because of the extinction of competitors post K-Pg boundary^20,44^. However, this similar set of forces may not be operating in other lichen-forming fungal lineages as mentioned in the previous section – the effect is lineage-specific and can be confounded by phylogenetic relatedness.

Our results reveal that not all lineages could have seized the opportunity created by major historical events and underwent rapid diversification. Similar findings have also been reported in a recent study focusing on frogs, where only the evolutionary histories of three lineages unravel accelerated diversification after the K-Pg event. There are clearly lineage-specific patterns of macroevolutionary dynamics demonstrated in many macroevolutionary studies^29^, and our results further imply that there can be lineage-specific effects of evolving different trait states on variations of macroevolutionary dynamics. Specifically, the evolution of the macrolichen phenotype might be correlated with an increase in diversification rate, but only in certain lineages, after the K-Pg boundary, although the pattern is not universal and can be explained by phylogenetic relatedness. Our results imply that although the macroevolutionary dynamics in lichen-forming fungi, and other organisms in general, could be contingent to evolutionary innovations, other factors pertaining to biological variation among lineages, variation in the nature of the putative evolutionary innovation among lineages – e.g., different growth forms exist in macrolichens – and even stochastic events may mask the effect from the previous events. Furthermore, micro-evolutionary process was not taken into account in our macro-evolutionary analyses. The generation time for example may differ between lineages, and especially the disparity in growth rate between micro- and macrolichens could be an innate difference leading to distinct evolutionary rate across lineages. Additionally, as mentioned before the K-Pg mass extinction event was not the only major geohistorical or biological event that might have affected the rate of diversification during the transition from Mesozoic to Cenozoic eras. For example, the KTR event (125 to 80 MYA) is characterized by rapid diversification of angiosperms and mammals^45,46^. The rapid organismal diversification, particularly in angiosperms, during KTR has been hypothesized impacted the global biodiversity pattern and might have directly affected the diversification rates of other evolutionary lineages^49^. Furthermore, the Paleocene-Eocene thermal maximum (55 MYA; PETM) could have also impacted the pattern of evolutionary diversification^50^. Changes in temperature and precipitation patterns can significantly affect the distribution of lichen-forming fungi. Given the high uncertainty in phylogenetic dating and the estimated phylogenetic position of rate shifts scatter across 100 to 40 MYA, we cannot rule out the effect of environmental and climatic changes occurred during late Cretaceous or early Cenozoic on the variation in diversification dynamics of lichen-forming fungi. Nevertheless, our study augments the growing evidence for major effects on re-shaping terrestrial biodiversity pattern by both biological (e.g., KTR) and geohistorical e.g., (K-Pg and PETM) events.

Future comparative studies that include comprehensive sampling and family-specific study design may help to tease apart the relative effects of the major historical events – i.e., KTR, K-Pg, and PETM – and evolutionary innovation on variation in biodiversity pattern across geological times and evolutionary lineages. Comparative studies that have extensive taxon sampling within each family can also help to test whether the power to detect changes in macroevolutionary dynamics is not only determined by the abundance of taxon sampling and missing sample adjustment, but also by the phylogenetic depth – e.g., including different genera, or families – of the investigated phylogeny. For example, studies that focus on within family diversification often infer a major change in the diversification dynamics during the Miocene, while those that focus on a larger and deeper scale phylogenetic history, such as this study, reveal a significant effect around the Paleocene^5^. That is, the most apparent pattern that can be identified from a macroevolution comparative study may vary depending on the scope of phylogenetic sampling and geological time.

It is worthy of noting that the methods to infer shifts in diversification pattern and trait-dependent diversification based on reconstructed molecular phylogeny are still in its infancy. Limitations of methods used are widely debated and controversies do exist^51,52^. We too found an inconsistent result between our study and a previous study using the same method, BAMM specifically. Teloschistaceae was shown to have experienced significant diversification rate acceleration around 100 MYA^40^. Our result, which also places the common ancestor of Teloschistaceae around 100 MYA (Fig. 1), does not support a rate shift at the same node (marginal shift probability = 0.0013). This inconsistency may result because of different strategies of phylogenetic taxon sampling and geological time among studies. For example, more microlichens families have been included in the current study than in the previously mentioned study focusing on Teloschistaceae. Similarly, as pointed out in the previous paragraph, most empirical data sets may not have enough statistical power to detect trait-dependent diversification pattern using STRAPP. We want to emphasize that we do not reject the idea that there can be more shifts in diversification rate on the phylogeny, as BAMM has been shown to underestimate the number of rate shift, and that the diversification rate can be dependent upon trait states in lichen-forming fungi, even though they are highly lineage-specific. We only reveal that their effect on the variation of macroevolutionary dynamics do not result in statistically detectable differences using currently available data. The non-significant results can, for example, be due to the lack of statistical power, and the hypothesis of evolving macrolichens leading to accelerated diversification and a rate shift at the common ancestor for Teloschistaceae can still be real patterns that require additional investigations.

## Conclusions

By assembling publicly accessible sequence data that have been accumulated over the past decades from major lineages of the lichen-forming fungi, we provide a novel example from a mutualistic system that is biologically, ecologically, and evolutionarily distinct from the intensively investigated vertebrate (e.g., birds and frogs), plant (e.g., eudicots and ferns), and insect (e.g., beetles) systems and broaden our understanding on not only the origins of biodiversity, but also the possible causes of variation in biodiversity pattern. Studies that focus on different organismal groups and different geological time scales often come to different conclusions. This inconsistency can be due to the use of different data sets and methodological approaches, but can also unravel real variations in historical processes and contingencies among organismal groups, as was revealed in our study by comparing the diversification dynamics between the two-main lichen-forming fungal subclasses, Lecanoromycetidae and Ostropomycetidae. Both subclasses persisted throughout major historical events, but only certain families prospered and rapidly diversified when provided with new opportunities. The differences in reaction to historical events might be related to the evolution of specific traits, but the currently available data, as well as limited statistical models, prevent us from making definitive inferences. While there is no doubt that the methodological approaches will continue to improve and more and more data are becoming available, the best way to understand how lineage-specific factors may lead to variation in the diversification pattern is to study more of them.

## Materials and Methods

### Phylogeny, the estimation of sampling completeness, and trait data

Recent molecular phylogenetic studies have gradually built a broad picture of the diversification history in different families and classes of lichen-forming fungi^40,41,53–56^. We used a recent time-calibrated phylogeny of Lecanoromycetes that included samples from most families and genera of the subclasses Lecanoromycetidae and Ostropomycetidae (details in^43^ for all the subsequent analyses and also used the classification proposed in this paper. We note that phylogenies inferred from more comprehensively sampled data would likely provide higher statistical power and robust results. However, our analyses are based on the most comprehensive phylogeny currently available.

Bias in taxonomic completeness overall across the phylogeny and between different trait states are the main source of uncertainty in large-scale analysis of biodiversity patterns, particularly in poorly known groups, such as lichen-forming fungi. We acknowledge that there have been several taxonomic studies on lichen-forming fungi, and many cryptic species have been uncovered in recent years, suggesting the potential for many additional undescribed species that are disproportionally distributed across lineages^57,58^. While accounting for unknown species is a challenge in any analysis, we relied on a recent account of families and genera in Ascomycota^42^ to estimate sampling bias in different genera (Supplementary Materials; frag_genus.txt).

Our morphological trait data were based primarily on the work of Jaklitsch *et al*.^42^. Specifically, we included all types of foliose and fruticose lichens in the character state “macrolichen”, whereas crustose species and species with small squamules were categorized as “microlichen”. A table of trait states for each of the sampled taxa can be found in Supplementary Materials (Oslec_states.csv).

### Macroevolutionary dynamics reconstruction

We estimated macroevolutionary rates and tested for their dependencies on morphological trait states using the program BAMM^59^. Our focus was on investigating support for lineage-specific and trait state differences in net diversification rate, given the difficulties in estimating extinction rates from molecular data^60^. One advantage of BAMM is that complex macroevolutionary mixture models are assessed with rate shifts across the tree, including accelerating and decelerating diversification rates. While trait-dependent diversification models are not directly fit to the tree, correlations between trait states and diversification rates can be assessed *post hoc* using STRAPP^61^. Therefore, we used BAMM to investigate evidence of accelerating or decelerating diversification through time and if the rate shifts were associated with critical geological events (e.g., the K-Pg boundary), and then to test for correlations between lichen growth forms (macro- versus microlichens) and net diversification rate.

Net-diversification, speciation and extinction rates through time based on^43^ were estimated using the program BAMM version 2.5. The initial values for speciation and extinction rates as well as the number of rate shift were estimated using the setBAMMpriors function in the R BAMMtools package^59^ and subsequently specified in the BAMM control file. A total of 2 × 10^8^ generations of rjMCMC searches with samples stored every 1 × 10^5^ generations were launched using the speciation-extinction mode. A total of 1000 post burnin samples (50%) were retained for the following analyses.

Using the posteriors generated from the rjMCMC searches, we aimed to (1) reconstruct the diversification history of Lecanoromycetidae and Ostropomycetidae, (2) test whether there is evidence of changes in diversification dynamics, particularly accelerating or decelerating diversification rate associated with major historical events, and (3) examine whether different growth forms in lichen-forming fungi are associated with different diversification rates. We visualized the reconstructed history of macroevolutionary dynamics with the plot.bammdata function from BAMMtools. We utilized marginal likelihood values to infer the phylogenetic position where diversification rate may have changed (accelerated/decelerated). We further plotted the diversification rate through time plots for each of the two subclasses and individual families that have a total species number greater than 500 (Table 1; family classification followed^43^ using the plotRateThroughTime function. We used the getTipRates function to extract the mean net-diversification rates at tips of the phylogeny, and then tested statistically whether species currently associated with different growth forms may exhibit different diversification rates. We also used the traitDependentBAMM [STRAPP^61^] function in BAMMtools to further investigate whether the net-diversification rate differs between species of different trait states while accounting for phylogenetic relatedness among taxa. The STRAPP analysis was performed with 1 × 10^4^ iterations and Spearman’s rho as test statistics. We also applied Pearson correlation as test statistics and Mann-Whitney test to assess whether our results were robust.

**Table 1:** Estimated number of species per family^42,43^.

Subclass	Order	Family	Number of Species
**Lecanoromycetidae**	Lecanorales	Parmeliaceae	2760*
		Gypsoplacaceae	1
		Tephromelataceae	50
		Lecanoraceae	**780**
		Pilocarpaceae	380
		Malmideaceae	53
		Cladoniaceae	**810**
		Ramalinaceae	**675**
		Biatoraceae	150
		Psoraceae	55
		Sphaerophoraceae	38
		Catillariaceae	175
		Scoliciosporaceae	16
		Psilolechiaceae	4
	Teloschistales	Teloschistaceae	**700**
		Megalosporaceae	38
		Brigantiaeaceae	50
	Caliciales	Physciaceae	**580**
		Caliciaceae	**630**
	Peltigerales	Peltigeraceae	**611**
		Massalongiaceae	5
		Vahliellaceae	8
		Koeberiaceae	8
		Collemataceae	190
		Placynthiaceae	30
		Pannariaceae	400
		Coccocarpiaceae	38
	Lecideales	Lecideaceae	200
	Rhizocarpales	Rhizocarpaceae	230
	Sporastatiales	Sporastatiaceae	5
**Ostropomycetidae**	Graphidales	Diploschistaceae	**600**
		Thelotremataceae	250
		Graphidaceae	**1250**
		Fissurinaceae	160
		Gomphillaceae	420
	Gyalectales	Porinaceae	360
		Coenogoniaceae	90
		Sagiolechiaceae	4
		Gyalectaceae	90
		Phlyctidaceae	20
	Odontotrematales	Odontotremataceae	22
	“Ostropales”	Asconditella clades	14
		Stictidaceae	210
		Ascarosporinaceae	5
	Thelenellales	Thelenellaceae	65
	Ochrolechiales	Ochrolechiaceae	60
		Varicellariaceae	7
		Variolariaceae	5
		Microcaliciaceae	4
		Megasporaceae	240
	Pertusariales	Pertusariaceae	400
		Agyriaceae	5
		Icmadophilaceae	55
		Coccotremataceae	26
	Baeomycetales	Trapeliaceae	117
		Xylographaceae	33
		Arctomiaceae	12
		Baeomycetaceae	16
		Cameroniaceae	2
		Protothelenellaceae	14
		Arthrorhaphidaceae	14
		Hymeneliaceae	28

*For families that contain more than 500 species the species numbers are shown in bold.

### The evolution of macro- and micro-lichens

To reconstruct the evolutionary history of macrolichens and microlichens, we fit discrete models (binary states) of character evolution using the ace function in the ape package^62^. Likelihood ratio tests were used to compare models that assume either equal or asymmetric rate of transition between trait states. The ancestral states at each node of the empirical tree were estimated using the selected model. To visualize the evolution of lichen growth forms, we further performed 100 stochastic character mappings on the empirical tree using the make.simmap function and plotted a summary of state probabilities for each branch with the densityMap function in the phytools package^63^.

## Supplementary information

Supplementary Dataset 1

Supplementary Dataset 2

## Data Availability

The studied phylogeny was retrieved from^43^. The sampling fraction and trait state files for BAMM analyses were appended as Supplementary Materials.
