# Supplementary material for: Accelerated diversifications in three diverse families of morphologically complex lichen-forming fungi link to major historical events
Source: Sci Rep. 2019 Jun 28;9:8518. doi: 10.1038/s41598-019-44881-1 (PMC6599062; doi:10.1038/s41598-019-44881-1)
Supplement: Supplementary file 1 — Supplementary Dataset 1 [file 41598_2019_44881_MOESM1_ESM.docx]

**Title:** Accelerated diversifications in three diverse families of morphologically complex lichen-forming fungi link to major historical events

**Authors:**

Jen-Pan Huang^1,4,a,*^, Ekaphan Kraichak^2,b^, Steven D. Leavitt^3,c^, Matthew P. Nelsen^1,d^, and H. Thorsten Lumbsch^1,e^

^1^Integrative Research Center, The Field Museum, Chicago, IL 60605, USA

^2^Department of Botany, Faculty of Science, Kasetsart University, Bangkok, Thailand

^3^Department of Biology and M. L. Bean Life Science Museum, Brigham Young University, Provo, UT 84602, USA

^4^Biodiversity Research Center, Academia Sinica, Taipei, Taiwan

a [jphuang@sinica.edu.tw](mailto:jhuang@fieldmuseum.org)

b [ekaphan.k@ku.th](mailto:ekaphan.k@ku.th)

c [steve_leavitt@byu.edu](mailto:steve_leavitt@byu.edu)

d [mnelsen@fieldmuseum.org](mailto:mnelsen@fieldmuseum.org)

e [tlumbsch@fieldmuseum.org](mailto:tlumbsch@fieldmuseum.org)

*Corresponding author

1.0

Acarospora_laqueata Acarospora 0.5

Pleopsidium_chlorophanum Pleopsidium 0.333

Arctomia_borbonica Arctomia 0.3

Arctomia_delicatula Arctomia 0.3

Arctomia_teretiuscula Arctomia 0.3

Gregorella_humida Gregorella 1

Wawea_fruticulosa Wawea 1

Arthrorhaphis_citrinella Anthrorhaphis 0.077

Ainoa_mooreana Ainoa 1

Baeomyces_heteromorphus Baeomyces 0.222

Baeomyces_rufus Baeomyces 0.222

Phyllobaeis_erythrella Phyllobaeis 0.4

Phyllobaeis_imbricata Phyllobaeis 0.4

Brigantiaea_fuscolutea Brigantiaea 0.069

Brigantiaea_leucoxantha Brigantiaea 0.069

Buellia_frigida Buellia 0.007

Buellia_stillingiana Buellia 0.007

Calicium_viride Calicium 0.04

Dimelaena_radiata Dimelaena 0.125

Pyxine_sorediata Pyxine 0.029

Pyxine_subcinerea Pyxine 0.029

Cameronia_pertusarioides Cameronia 1

Cameronia_tecta Cameronia 1

Carbonicola_anthracophila Carbonicola 0.667

Hypocenomyce_castaneocinerea Carbonicola 0.667

Catillaria_contristans Catillaria 0.007

Cladia_aggregata Cladia 0.4

Cladia_corallaizon Cladia 0.4

Cladia_deformis Cladia 0.4

Cladia_dumicola Cladia 0.4

Cladia_ferdinandii Cladia 0.4

Cladia_inflata Cladia 0.4

Cladia_moniliformis Cladia 0.4

Cladia_retipora Cladia 0.4

Cladia_schizopora Cladia 0.4

Cladia_sullivanii Cladia 0.4

Cladonia_caroliniana Cladonia 0.018

Cladonia_digitata Cladonia 0.018

Cladonia_mitis Cladonia 0.018

Cladonia_peziziformis Cladonia 0.018

Cladonia_rangiferina Cladonia 0.018

Cladonia_stipitata Cladonia 0.018

Cladonia_sulcata Cladonia 0.018

Heterodea_beaugleholei Cladonia 0.018

Heterodea_muelleri Cladonia 0.018

Metus_conglomeratus Metus 0.333

Pycnothelia_papillaria Pycnothelia 0.5

Ramalinora_glaucolivida Ramalinora 1

Coccocarpia_domingensis Coccocarpia 0.1

Coccocarpia_erythroxyli Coccocarpia 0.1

Coccocarpia_palmicola Coccocarpia 0.1

Spilonemella_americana Spilonemella 0.5

Coccotrema_cucurbitula Coccotrema 0.25

Coccotrema_maritimum Coccotrema 0.25

Coccotrema_pocillarium Coccotrema 0.25

Gyalectaria_diluta Coccotrema 0.25

Gyalectaria_gyalectoides Gyalectaria 0.333

Coenogonium_leprieurii Coenogonium 0.033

Coenogonium_luteum Coenogonium 0.033

Coenogonium_pineti Coenogonium 0.033

Collema_cristatum Collema 0.15

Collema_furfuraceum Collema 0.15

Collema_nigrescens Collema 0.15

Collema_parvum Collema 0.15

Collema_subconveniens Collema 0.15

Collema_undulatum Collema 0.15

Leptogium_azureum Leptogium 0.114

Leptogium_cyanescens Leptogium 0.114

Leptogium_diffractum Leptogium 0.114

Leptogium_furfuraceum Leptogium 0.114

Leptogium_lichenoides Leptogium 0.114

Leptogium_plicatile Leptogium 0.114

Leptogium_pseudofurfuraceum Leptogium 0.114

Leptogium_saturninum Leptogium 0.114

Echinoplaca_epiphylla Echinoplaca 0.025

Gomphillus_calycioides Gomphillus 0.167

Gyalidea_fritzei Gyalidea 0.02

Gyalideopsis_vulgaris Gyalideopsis 0.0105

Astrochapsa_astroidea Astrochapsa 0.045

Carbacanthographis_stictica Carbacanthographis 0.036

Chapsa_alborosella Chapsa 0.131

Chapsa_indica Chapsa 0.131

Chapsa_leprocarpa Chapsa 0.131

Chapsa_niveocarpa Chapsa 0.131

Chapsa_patens Chapsa 0.131

Chapsa_phlyctidioides Chapsa 0.131

Chapsa_pulchra Chapsa 0.131

Chapsa_sublilacina Chapsa 0.131

Crutarndina_petractoides Crutardina 1

Diorygma_poitaei Diorygma 0.014

Diploschistes_actinostomus Diploschistes 0.226

Diploschistes_cinereocaesius Diploschistes 0.226

Diploschistes_elixii Diploschistes 0.226

Diploschistes_euganeus Diploschistes 0.226

Diploschistes_muscorum Diploschistes 0.226

Diploschistes_scruposus Diploschistes 0.226

Diploschistes_sticticus Diploschistes 0.226

Dyplolabia_afzelii Dyplolabia 0.25

Fissurina_insidiosa Fissurina 0.006

Glaucotrema_glaucophaeum Glaucotrema 0.25

Glyphis_substriatula Glyphis 0.143

Graphis_scripta Graphis 0.002

Leucodecton_subcompunctum Leucodecton 0.036

Myriochapsa_psoromica Myriochapsa 0.333

Myriotrema_olivaceum Myriotrema 0.015

Ocellularia_allosporoides Ocellularia 0.008

Ocellularia_garoana Ocellularia 0.008

Ocellularia_profunda Ocellularia 0.008

Phaeographis_intricans Phaeographis 0.006

Platythecium_grammitis Platythecium 0.038

Pycnotrema_pycnoporellum Pycnotrema 0.333

Rhabdodiscus_subcavatus Rhabdodiscus 0.024

Stegobolus_anamorphus Stegobolus 0.067

Thelotrema_adjectum Thelotrema 0.066

Thelotrema_diplotrema Thelotrema 0.066

Thelotrema_lepadinum Thelotrema 0.066

Thelotrema_porinaceum Thelotrema 0.066

Thelotrema_porinoides Thelotrema 0.066

Thelotrema_subtile Thelotrema 0.066

Thelotrema_suecicum Thelotrema 0.066

Topeliopsis_acutispora Topeliopsis 0.2

Topeliopsis_decorticans Topeliopsis 0.2

Topeliopsis_muscigena Topeliopsis 0.2

Topeliopsis_subdenticulata Topeliopsis 0.2

Wirthiotrema_glaucopallens Wirthiotrema 0.143

Belonia_russula Gyalecta 0.12

Gyalecta_flotowii Gyalecta 0.12

Gyalecta_hypoleuca Gyalecta 0.12

Gyalecta_jenensis Gyalecta 0.12

Gyalecta_truncigena Gyalecta 0.12

Gyalecta_ulmi Gyalecta 0.12

Gypsoplaca_macrophylla Gypsoplaca 1

Hymenelia_epulotica Hymenelia 0.08

Hymenelia_lacustris Hymenelia 0.08

Tremolecia_atrata Tremolecia 0.167

Ainoa_geochroa Ainoa 0.154

Dibaeis_baeomyces Dibaeis 0.154

Icmadophila_ericetorum Icmadophila 0.25

Siphula_ceratites Siphula 0.038

Thamnolia_subuliformis Thamnolia 0.5

Thamnolia_vermicularis Thamnolia 0.5

Steinera_glaucella Steinera 0.25

Lecanora_achroa Lecanora 0.017

Lecanora_caesiorubella Lecanora 0.017

Lecanora_contractula Lecanora 0.017

Lecanora_flavopallida Lecanora 0.017

Lecanora_garovaglii Lecanora 0.017

Lecanora_hybocarpa Lecanora 0.017

Lecanora_muralis Lecanora 0.017

Lecanora_symmicta Lecanora 0.017

Lecanora_tropica Lecanora 0.017

Lecanora_vainioi Lecanora 0.017

Lecidella_carpathica Lecidella 0.063

Lecidella_elaeochroma Lecidella 0.063

Lecidella_euphorea Lecidella 0.063

Lecidella_patavina Lecidella 0.063

Lecidella_stigmatea Lecidella 0.063

Rhizoplaca_chrysoleuca Rhizoplaca 0.545

Rhizoplaca_haydenii Rhizoplaca 0.545

Rhizoplaca_melanophthalma Rhizoplaca 0.545

Rhizoplaca_parilis Rhizoplaca 0.545

Rhizoplaca_porterii Rhizoplaca 0.545

Rhizoplaca_shushanii Rhizoplaca 0.545

Lecidea_cyrtidia Lecidea 0.06

Lecidea_auriculata Lecidea 0.06

Lecidea_fuscoatra Lecidea 0.06

Lecidea_laboriosa Lecidea 0.06

Lecidea_plana Lecidea 0.06

Lecidea_silacea Lecidea 0.06

Porpidia_albocaerulescens Porpidia 0.067

Porpidia_speirea Porpidia 0.067

Letrouitia_domingensis Letroutia 0.167

Letrouitia_parabola Letroutia 0.167

Letrouitia_vulpina Letroutia 0.167

Lobaria_hallii Lobaria 0.05

Lobaria_pulmonaria Lobaria 0.05

Lobaria_virens Lobaria 0.05

Lobariella_pallida Lobariella 0.029

Sticta_beauvoisii Sticta 0.01

Sticta_canariensis Sticta 0.01

Savoronala_madagascariensis Malmidea 0.02

Leptochidium_albociliatum Leptochidium 0.5

Massalongia_carnosa Massalongia 0.5

Polychidium_muscicola Polychidium 1

Austroblastenia_pauciseptata Austroblastenia 1

Austroblastenia_pupa Austroblastenia 1

Megaloblastenia_marginiflexa Megaloblastenia 0.5

Megalospora_austropacifica Megalospora 0.242

Megalospora_coccodes Megalospora 0.242

Megalospora_disjuncta Megalospora 0.242

Megalospora_lopadioides Megalospora 0.242

Megalospora_pulverata Megalospora 0.242

Megalospora_subtuberculosa Megalospora 0.242

Megalospora_sulphurata Megalospora 0.242

Megalospora_tuberculosa Megalospora 0.242

Sipmaniella_sulphureofusca Sipmaniela 1

Aspicilia_caesiocinerea Aspicilia 0.015

Aspicilia_cinerea Aspicilia 0.015

Aspicilia_desertorum Aspicilia 0.015

Circinaria_contorta Circinaria 0.08

Circinaria_hispida Circinaria 0.08

Lobothallia_radiosa Lobothalia 0.111

Microcalicium_ahlneri Microcalicium 0.75

Microcalicium_arenarium Microcalicium 0.75

Microcalicium_disseminatum Microcalicium 0.75

Miltidea_ceroplasta Miltidea 1

Nephroma_antarcticum Nephroma 0.528

Nephroma_arcticum Nephroma 0.528

Nephroma_areolatum Nephroma 0.528

Nephroma_bellum Nephroma 0.528

Nephroma_cellulosum Nephroma 0.528

Nephroma_expallidum Nephroma 0.528

Nephroma_foliolatum Nephroma 0.528

Nephroma_helveticum Nephroma 0.528

Nephroma_hensseniae Nephroma 0.528

Nephroma_laevigatum Nephroma 0.528

Nephroma_occultum Nephroma 0.528

Nephroma_parile Nephroma 0.528

Nephroma_plumbeum Nephroma 0.528

Nephroma_resupinatum Nephroma 0.528

Nephroma_skottsbergii Nephroma 0.528

Nephroma_sulcatum Nephroma 0.528

Nephroma_tangeriense Nephroma 0.528

Nephroma_tropicum Nephroma 0.528

Nephroma_venosum Nephroma 0.528

Anzina_carneonivea Anzia 1

Puttea_margaritella Puttea 0.333

Ochrolechia_androgyna Ochrolechia 0.1

Ochrolechia_parella Ochrolechia 0.1

Ochrolechia_peruensis Ochrolechia 0.1

Ochrolechia_subpallescens Ochrolechia 0.1

Ochrolechia_upsaliensis Ochrolechia 0.1

Ochrolechia_yasudae Ochrolechia 0.1

Pertusaria_amara Pertusaria 0.018

Pertusaria_corallina Pertusaria 0.018

Pertusaria_dactylina Pertusaria 0.018

Pertusaria_hemisphaerica Pertusaria 0.018

Pertusaria_lactea Pertusaria 0.018

Pertusaria_scaberula Pertusaria 0.018

Pertusaria_subventosa Pertusaria 0.018

Varicellaria_culbersonii Varicellaria 0.429

Varicellaria_rhodocarpa Varicellaria 0.429

Varicellaria_velata Varicellaria 0.429

Odontotrema_phacidiellum Odontotrema 0.286

Odontotrema_phacidioides Odontotrema 0.286

Degelia_plumbea Degelia 0.045

Erioderma_verruculosum Erioderma 0.025

Fuscopannaria_ignobilis Fuscopannaria 0.017

Pannaria_rubiginosa Pannaria 0.04

Parmeliella_triptophylla Pannaria 0.04

Staurolemma_omphalarioides Staurolemma 0.333

Alectoria_ochroleuca Alectoria 0.222

Alectoria_sarmentosa Alectoria 0.222

Allocetraria_flavonigrescens Alloletraria 0.111

Austroparmelina_macrospora Austroparmelia 0.154

Austroparmelina_pruinata Austroparmelia 0.154

Brodoa_intestiniformis Brodoa 0.333

Bryocaulon_divergens Bryocaulon 0.25

Bryoria_americana Bryoria 0.22

Bryoria_capillaris Bryoria 0.22

Bryoria_fremontii Bryoria 0.22

Bryoria_furcellata Bryoria 0.22

Bryoria_fuscescens Bryoria 0.22

Bryoria_glabra Bryoria 0.22

Bryoria_implexa Bryoria 0.22

Bryoria_nadvornikiana Bryoria 0.22

Bryoria_simplicior Bryoria 0.22

Bryoria_smithii Bryoria 0.22

Bryoria_trichodes Bryoria 0.22

Cetraria_islandica Cetraria 0.267

Cetraria_kamtczatica Cetraria 0.267

Cetraria_nigricans Cetraria 0.267

Cetraria_sepincola Cetraria 0.267

Cetrariella_commixta Cetrariella 0.75

Cetrariella_delisei Cetrariella 0.75

Cetrariella_fastigiata Cetrariella 0.75

Dactylina_arctica Dactylina 0.5

Emodomelanelia_masonii Emodomelanelia 1

Evernia_prunastri Evernia 0.1

Flavocetraria_cucullata Flavocetraria 0.667

Flavocetraria_nivalis Flavocetraria 0.667

Flavoparmelia_caperata Flavoparmelia 0.063

Flavoparmelia_soredians Flavoparmelia 0.063

Flavopunctelia_flaventior Flavopunctelia 0.2

Hypogymnia_physodes Hypogymnia 0.011

Hypotrachyna_caraccensis Hypotrachyna 0.008

Hypotrachyna_degelii Hypotrachyna 0.008

Imshaugia_aleurites Imshaugia 0.143

Letharia_columbiana Letharia 0.167

Masonhalea_richardsonii Masonhalea 0.5

Melanelia_hepatizon Melanelia 0.2

Melanelixia_albertana Melanelixia 0.533

Melanelixia_californica Melanelixia 0.533

Melanelixia_fuliginosa Melanelixia 0.533

Melanelixia_glabratuloides Melanelixia 0.533

Melanelixia_glabroides Melanelixia 0.533

Melanelixia_subaurifera Melanelixia 0.533

Melanelixia_subglabra Melanelixia 0.533

Melanelixia_villosella Melanelixia 0.533

Melanohalea_elegantula Melanohalea 0.636

Melanohalea_exasperata Melanohalea 0.636

Melanohalea_exasperatula Melanohalea 0.636

Melanohalea_gomukhensis Melanohalea 0.636

Melanohalea_halei Melanohalea 0.636

Melanohalea_infumata Melanohalea 0.636

Melanohalea_laciniatula Melanohalea 0.636

Melanohalea_multispora Melanohalea 0.636

Melanohalea_olivacea Melanohalea 0.636

Melanohalea_poeltii Melanohalea 0.636

Melanohalea_septentrionalis Melanohalea 0.636

Melanohalea_subelegantula Melanohalea 0.636

Melanohalea_subolivacea Melanohalea 0.636

Melanohalea_trabeculata Melanohalea 0.636

Menegazzia_terebrata Menegazzia 0.014

Montanelia_disjuncta Montanelia 0.8

Montanelia_panniformis Montanelia 0.8

Montanelia_sorediata Montanelia 0.8

Montanelia_tominii Montanelia 0.8

Myelochroa_aurulenta Myelochroa 0.067

Myelochroa_irrugans Myelochroa 0.067

Nephromopsis_leucostigma Nephromopsis 0.05

Parmelia_saxatilis Parmelia 0.04

Parmelia_serrana Parmelia 0.04

Parmelina_tiliacea Parmelina 0.1

Parmeliopsis_ambigua Parmeliopsis 0.667

Parmeliopsis_hyperopta Parmeliopsis 0.667

Parmotrema_austrosinense Parmotrema 0.013

Parmotrema_reticulatum Parmotrema 0.013

Parmotrema_subtinctorium Parmotrema 0.013

Parmotrema_tinctorum Parmotrema 0.013

Platismatia_glauca Platismatia 0.5

Pleurosticta_acetabulum Pleurosticta 0.5

Protoparmelia_badia Protoparmelia 0.029

Protousnea_magellanica Protousnea 0.125

Pseudephebe_pubescens Pseudephebe 0.5

Pseudevernia_consocians Pseudevernia 0.5

Pseudevernia_furfuracea Pseudevernia 0.5

Punctelia_rudecta Punctelia 0.022

Relicina_sydneyensis Relicina 0.019

Tuckermannopsis_chlorophylla Tuckermannopsis 0.222

Tuckermannopsis_ciliaris Tuckermannopsis 0.222

Usnea_antarctica Usnea 0.031

Usnea_ceratina Usnea 0.031

Usnea_cornuta Usnea 0.031

Usnea_florida Usnea 0.031

Usnea_glabrescens Usnea 0.031

Usnea_lapponica Usnea 0.031

Usnea_silesiaca Usnea 0.031

Usnea_sphacelata Usnea 0.031

Usnea_strigosa Usnea 0.031

Usnea_subfloridana Usnea 0.031

Usnea_wasmuthii Usnea 0.031

Usnocetraria_oakesiana Usnocetaria 1

Vulpicida_canadensis Vulpicida 0.833

Vulpicida_juniperinus Vulpicida 0.833

Vulpicida_pinastri Vulpicida 0.833

Vulpicida_tubulosus Vulpicida 0.833

Vulpicida_viridis Vulpicida 0.833

Xanthoparmelia_chlorochroa Xanthoparmelia 0.009

Xanthoparmelia_conspersa Xanthoparmelia 0.009

Xanthoparmelia_lipochlorochroa Xanthoparmelia 0.009

Xanthoparmelia_mougeotii Xanthoparmelia 0.009

Xanthoparmelia_saxeti Xanthoparmelia 0.009

Xanthoparmelia_tinctina Xanthoparmelia 0.009

Xanthoparmelia_wyomingica Xanthoparmelia 0.009

Peltigera_aphthosa Peltigera 0.07

Peltigera_canina Peltigera 0.07

Peltigera_degenii Peltigera 0.07

Peltigera_horizontalis Peltigera 0.07

Peltigera_leucophlebia Peltigera 0.07

Peltigera_membranacea Peltigera 0.07

Peltigera_praetextata Peltigera 0.07

Solorina_crocea Solorina 0.2

Solorina_saccata Solorina 0.2

Agyrium_rufum Agyrium 1

Loxosporopsis_corallifera Loxosporopsis 1

Pertusaria_gibberosa Pertusaria 0.015

Pertusaria_hermaka Pertusaria 0.015

Pertusaria_leioplaca Pertusaria 0.015

Pertusaria_paramerae Pertusaria 0.015

Pertusaria_pertusa Pertusaria 0.015

Pertusaria_pustulata Pertusaria 0.015

Phlyctis_agelaea Phlyctis 0.1

Phlyctis_argena Phlyctis 0.1

Anaptychia_palmatula Anaptychia 0.067

Heterodermia_vulgaris Heterodermia 0.01

Phaeophyscia_orbicularis Phaeophyscia 0.033

Physcia_aipolia Physcia 0.25

Physcia_dubia Physcia 0.25

Physconia_muscigena Physconia 0.04

Rinodina_tephraspis Rinodina 0.003

Byssoloma_leucoblepharum Byssoloma 0.047

Byssoloma_subdiscordans Byssoloma 0.047

Calopadia_foliicola Calopadia 0.04

Fellhanera_bouteillei Felhanera 0.014

Micarea_alabastrites Micarea 0.03

Micarea_denigrata Micarea 0.03

Micarea_sylvicola Micarea 0.03

Nelsenium_usnicum Nelsenium 1

Placynthium_flabellosum Placynthium 0.16

Placynthium_nigrum Placynthium 0.16

Placynthium_pannariellum Placynthium 0.16

Placynthium_tantaleum Placynthium 0.16

Porina_aenea Porina 0.036

Porina_byssophila Porina 0.036

Porina_epiphylla Porina 0.036

Porina_lectissima Porina 0.036

Porina_nitidula Porina 0.036

Protothelenella_corrosa Protothelenella 0.182

Protothelenella_sphinctrinoidella Protothelenella 0.182

Psilolechia_leprosa Psilolechia 0.5

Psilolechia_lucida Psilolechia 0.5

Protoblastenia_calva Protoblastenia 0.071

Psora_decipiens Psora 0.057

Psora_rubiformis Psora 0.057

Mycobilimbia_tetramera Mycobilimbia 0.2

Byssolecania_variabilis Byssolecania 0.167

Micarea_adnata Micarea 1

Bacidia_rosella Bacidia 0.009

Bacidia_schweinitzii Bacidia 0.009

Bacidina_arnoldiana Bacidina 0.083

Biatora_subduplex Biatora 0.024

Bilimbia_sabuletorum Bilimbia 0.167

Crocynia_pyxinoides Crocynia 0.333

Herteliana_taylorii Herteliana 0.333

Lecania_atrynoides Lacania 0.04

Lecania_cyrtella Lacania 0.04

Lopezaria_versicolor Lopezaria 0.5

Niebla_cephalota Ramalina 0.016

Ramalina_complanata Ramalina 0.016

Ramalina_farinacea Ramalina 0.016

Ramalina_fastigiata Ramalina 0.016

Toninia_cinereovirens Toninia 0.012

Catolechia_wahlenbergii Catolechia 1

Rhizocarpon_disporum Rhizocarpon 0.031

Rhizocarpon_geminatum Rhizocarpon 0.031

Rhizocarpon_geographicum Rhizocarpon 0.031

Rhizocarpon_hochstetteri Rhizocarpon 0.031

Rhizocarpon_oederi Rhizocarpon 0.031

Rhizocarpon_sphaerosporum Rhizocarpon 0.031

Rhizocarpon_superficiale Rhizocarpon 0.031

Rhexophiale_rhexoblephara Rhexophiale 1

Sagiolechia_protuberans Sagiolechia 0.333

Loxospora_cismonica Loxospora 0.444

Loxospora_elatina Loxospora 0.444

Loxospora_lecanoriformis Loxospora 0.444

Loxospora_ochrophaea Loxospora 0.444

Schaereria_corticola Schaereria 0.188

Schaereria_dolodes Schaereria 0.188

Schaereria_fuscocinerea Schaereria 0.188

Scoliciosporum_intrusum Scoliciosporum 0.133

Scoliciosporum_umbrinum Scoliciosporum 0.133

Calycidium_cuneatum Calycidium 0.5

Neophyllis_melacarpa Neophyllis 0.5

Sphaerophorus_fragilis Sphaerophorus 0.25

Sphaerophorus_globosus Sphaerophorus 0.25

Sporastatia_polyspora Sporastatia 0.5

Sporastatia_testudinea Sporastatia 0.5

Toensbergia_leucococca Toensbergia 1

Lepraria_bergensis Lepraria 0.038

Lepraria_incana Lepraria 0.038

Lepraria_lobificans Lepraria 0.038

Squamarina_cartilaginea Squamarina 0.12

Squamarina_gypsacea Squamarina 0.12

Squamarina_lentigera Squamarina 0.12

Stereocaulon_paschale Stereocaulon 0.021

Stereocaulon_pileatum Stereocaulon 0.021

Stereocaulon_tomentosum Stereocaulon 0.021

Absconditella_lignicola Absconditella 0.167

Absconditella_sphagnorum Absconditella 0.167

Acarosporina_microspora Acarosporina 0.2

Cryptodiscus_gloeocapsa Cryptodiscus 0.111

Cyanodermella_viridula Cyanodermella 0.5

Schizoxylon_albescens Schizoxylon 0.029

Stictis_populorum Stictis 0.014

Caloplaca_atroflava Caloplaca 0.029

Caloplaca_chilensis Caloplaca 0.029

Caloplaca_chlorina Caloplaca 0.029

Caloplaca_cinnamomea Caloplaca 0.029

Caloplaca_conversa Caloplaca 0.029

Caloplaca_granulosa Caloplaca 0.029

Caloplaca_isidiigera Caloplaca 0.029

Caloplaca_polycarpa Caloplaca 0.029

Caloplaca_teicholyta Caloplaca 0.029

Caloplaca_variabilis Caloplaca 0.029

Gyalolechia_fulgens Gyalolechia 0.067

Gyalolechia_stipitata Gyalolechia 0.067

Niorma_chrysophthalma Niorma 1

Polycauliona_impolita Polycauliona 0.04

Pyrenodesmia_chalybaea Pyrenodesmia 0.167

Rufoplaca_scotoplaca Rufoplaca 0.167

Seirophora_californica Seirophora 0.182

Seirophora_lacunosa Seirophora 0.182

Sirenophila_eos Sirenophila 0.143

Solitaria_chrysophthalma Solitaria 1

Stellarangia_elegantissima Stellarangia 0.333

Teloschistes_exilis Teloschistes 0.15

Teloschistes_flavicans Teloschistes 0.15

Teloschistes_hosseusianus Teloschistes 0.15

Usnochroma_carphinea Usnochroma 1

Usnochroma_scoriophila Usnochroma 1

Variospora_thallincola Variospora 0.167

Variospora_velana Variospora 0.167

Wetmoreana_decipioides Wetmoreana 0.333

Xanthomendoza_fallax Xanthomendoza 0.25

Xanthomendoza_oregana Xanthomendoza 0.25

Xanthomendoza_poeltii Xanthomendoza 0.25

Xanthomendoza_trachyphylla Xanthomendoza 0.25

Xanthomendoza_weberi Xanthomendoza 0.25

Xanthopeltis_rupicola Xanthopeltis 1

Xanthoria_aureola Xanthoria 0.3

Xanthoria_calcicola Xanthoria 0.3

Xanthoria_parietina Xanthoria 0.3

Calvitimela_armeniaca Calvitimela 0.125

Lecidea_aglaea Lec 1

Mycoblastus_affinis Mycoblastus 0.2

Mycoblastus_sanguinarius Mycoblastus 0.2

Tephromela_atra Tephromela 0.067

Tephromela_grumosa Tephromela 0.067

Violella_fucata Violella 0.5

Chromatochlamys_muscorum Chromatochlamys 0.333

Thelenella_antarctica Thelenella 0.033

Thrombium_epigaeum Thrombium 0.2

Placopsis_cribellans Placopsis 0.033

Placopsis_perrugosa Placopsis 0.033

Placynthiella_icmalea Placynthiella 0.143

Trapelia_coarctata Trapelia 0.077

Trapeliopsis_granulosa Trapeliopsis 0.05

Umbilicaria_hyperborea Umbilicaria 0.143

Vahliella_californica Vahliella 0.375

Vahliella_leucophaea Vahliella 0.375

Vahliella_saubinetii Vahliella 0.375

Lithographa_tesserata lithographa 0.1

Xylographa_trunciseda Xylographa 0.1

Xylographa_vitiligo Xylographa 0.1
