# Supplementary material for: Accelerated diversifications in three diverse families of morphologically complex lichen-forming fungi link to major historical events
Source: Sci Rep. 2019 Jun 28;9:8518. doi: 10.1038/s41598-019-44881-1 (PMC6599062; doi:10.1038/s41598-019-44881-1)
Supplement: Supplementary file 2 — Supplementary Dataset 2 [file 41598_2019_44881_MOESM2_ESM.docx]

**Title:** Accelerated diversifications in three diverse families of morphologically complex lichen-forming fungi link to major historical events

**Authors:**

Jen-Pan Huang^1,4,a,*^, Ekaphan Kraichak^2,b^, Steven D. Leavitt^3,c^, Matthew P. Nelsen^1,d^, and H. Thorsten Lumbsch^1,e^

^1^Integrative Research Center, The Field Museum, Chicago, IL 60605, USA

^2^Department of Botany, Faculty of Science, Kasetsart University, Bangkok, Thailand

^3^Department of Biology and M. L. Bean Life Science Museum, Brigham Young University, Provo, UT 84602, USA

^4^Biodiversity Research Center, Academia Sinica, Taipei, Taiwan

a [jphuang@sinica.edu.tw](mailto:jhuang@fieldmuseum.org)

b [ekaphan.k@ku.th](mailto:ekaphan.k@ku.th)

c [steve_leavitt@byu.edu](mailto:steve_leavitt@byu.edu)

d [mnelsen@fieldmuseum.org](mailto:mnelsen@fieldmuseum.org)

e [tlumbsch@fieldmuseum.org](mailto:tlumbsch@fieldmuseum.org)

*Corresponding author

names,state

Acarospora_laqueata,0

Pleopsidium_chlorophanum,0

Arctomia_borbonica,0

Arctomia_delicatula,0

Arctomia_teretiuscula,0

Gregorella_humida,0

Wawea_fruticulosa,0

Arthrorhaphis_citrinella,0

Ainoa_mooreana,0

Baeomyces_heteromorphus,0

Baeomyces_rufus,1

Phyllobaeis_erythrella,1

Phyllobaeis_imbricata,1

Brigantiaea_fuscolutea,0

Brigantiaea_leucoxantha,0

Buellia_frigida,0

Buellia_stillingiana,0

Calicium_viride,0

Dimelaena_radiata,0

Pyxine_sorediata,1

Pyxine_subcinerea,1

Cameronia_pertusarioides,0

Cameronia_tecta,0

Carbonicola_anthracophila,1

Hypocenomyce_castaneocinerea,1

Catillaria_contristans,0

Cladia_aggregata,1

Cladia_corallaizon,1

Cladia_deformis,1

Cladia_dumicola,1

Cladia_ferdinandii,1

Cladia_inflata,1

Cladia_moniliformis,1

Cladia_retipora,1

Cladia_schizopora,1

Cladia_sullivanii,1

Cladonia_caroliniana,1

Cladonia_digitata,1

Cladonia_mitis,1

Cladonia_peziziformis,1

Cladonia_rangiferina,1

Cladonia_stipitata,1

Cladonia_sulcata,1

Heterodea_beaugleholei,1

Heterodea_muelleri,1

Metus_conglomeratus,1

Pycnothelia_papillaria,1

Ramalinora_glaucolivida,1

Coccocarpia_domingensis,1

Coccocarpia_erythroxyli,1

Coccocarpia_palmicola,1

Spilonemella_americana,1

Coccotrema_cucurbitula,0

Coccotrema_maritimum,0

Coccotrema_pocillarium,0

Gyalectaria_diluta,0

Gyalectaria_gyalectoides,0

Coenogonium_leprieurii,1

Coenogonium_luteum,1

Coenogonium_pineti,1

Collema_cristatum,1

Collema_furfuraceum,1

Collema_nigrescens,1

Collema_parvum,1

Collema_subconveniens,1

Collema_undulatum,1

Leptogium_azureum,1

Leptogium_cyanescens,1

Leptogium_diffractum,1

Leptogium_furfuraceum,1

Leptogium_lichenoides,1

Leptogium_plicatile,1

Leptogium_pseudofurfuraceum,1

Leptogium_saturninum,1

Echinoplaca_epiphylla,0

Gomphillus_calycioides,0

Gyalidea_fritzei,0

Gyalideopsis_vulgaris,0

Astrochapsa_astroidea,0

Carbacanthographis_stictica,0

Chapsa_alborosella,0

Chapsa_indica,0

Chapsa_leprocarpa,0

Chapsa_niveocarpa,0

Chapsa_patens,0

Chapsa_phlyctidioides,0

Chapsa_pulchra,0

Chapsa_sublilacina,0

Crutarndina_petractoides,0

Diorygma_poitaei,0

Diploschistes_actinostomus,0

Diploschistes_cinereocaesius,0

Diploschistes_elixii,0

Diploschistes_euganeus,0

Diploschistes_muscorum,0

Diploschistes_scruposus,0

Diploschistes_sticticus,0

Dyplolabia_afzelii,0

Fissurina_insidiosa,0

Glaucotrema_glaucophaeum,0

Glyphis_substriatula,0

Graphis_scripta,0

Leucodecton_subcompunctum,0

Myriochapsa_psoromica,0

Myriotrema_olivaceum,0

Ocellularia_allosporoides,0

Ocellularia_garoana,0

Ocellularia_profunda,0

Phaeographis_intricans,0

Platythecium_grammitis,0

Pycnotrema_pycnoporellum,0

Rhabdodiscus_subcavatus,0

Stegobolus_anamorphus,0

Thelotrema_adjectum,0

Thelotrema_diplotrema,0

Thelotrema_lepadinum,0

Thelotrema_porinaceum,0

Thelotrema_porinoides,0

Thelotrema_subtile,0

Thelotrema_suecicum,0

Topeliopsis_acutispora,0

Topeliopsis_decorticans,0

Topeliopsis_muscigena,0

Topeliopsis_subdenticulata,0

Wirthiotrema_glaucopallens,0

Belonia_russula,0

Gyalecta_flotowii,0

Gyalecta_hypoleuca,0

Gyalecta_jenensis,0

Gyalecta_truncigena,0

Gyalecta_ulmi,0

Gypsoplaca_macrophylla,1

Hymenelia_epulotica,0

Hymenelia_lacustris,0

Tremolecia_atrata,0

Ainoa_geochroa,0

Dibaeis_baeomyces,1

Icmadophila_ericetorum,0

Siphula_ceratites,1

Thamnolia_subuliformis,1

Thamnolia_vermicularis,1

Steinera_glaucella,1

Lecanora_achroa,1

Lecanora_caesiorubella,1

Lecanora_contractula,1

Lecanora_flavopallida,1

Lecanora_garovaglii,1

Lecanora_hybocarpa,1

Lecanora_muralis,1

Lecanora_symmicta,1

Lecanora_tropica,1

Lecanora_vainioi,1

Lecidella_carpathica,0

Lecidella_elaeochroma,0

Lecidella_euphorea,0

Lecidella_patavina,0

Lecidella_stigmatea,0

Rhizoplaca_chrysoleuca,1

Rhizoplaca_haydenii,1

Rhizoplaca_melanophthalma,1

Rhizoplaca_parilis,1

Rhizoplaca_porterii,1

Rhizoplaca_shushanii,1

Lecidea_cyrtidia,0

Lecidea_auriculata,0

Lecidea_fuscoatra,0

Lecidea_laboriosa,0

Lecidea_plana,0

Lecidea_silacea,0

Porpidia_albocaerulescens,0

Porpidia_speirea,0

Letrouitia_domingensis,0

Letrouitia_parabola,0

Letrouitia_vulpina,0

Lobaria_hallii,1

Lobaria_pulmonaria,1

Lobaria_virens,1

Lobariella_pallida,1

Sticta_beauvoisii,1

Sticta_canariensis,1

Savoronala_madagascariensis,1

Leptochidium_albociliatum,1

Massalongia_carnosa,1

Polychidium_muscicola,1

Austroblastenia_pauciseptata,0

Austroblastenia_pupa,0

Megaloblastenia_marginiflexa,0

Megalospora_austropacifica,0

Megalospora_coccodes,0

Megalospora_disjuncta,0

Megalospora_lopadioides,0

Megalospora_pulverata,0

Megalospora_subtuberculosa,0

Megalospora_sulphurata,0

Megalospora_tuberculosa,0

Sipmaniella_sulphureofusca,0

Aspicilia_caesiocinerea,0

Aspicilia_cinerea,0

Aspicilia_desertorum,0

Circinaria_contorta,0

Circinaria_hispida,0

Lobothallia_radiosa,1

Microcalicium_ahlneri,0

Microcalicium_arenarium,0

Microcalicium_disseminatum,0

Miltidea_ceroplasta,0

Nephroma_antarcticum,1

Nephroma_arcticum,1

Nephroma_areolatum,1

Nephroma_bellum,1

Nephroma_cellulosum,1

Nephroma_expallidum,1

Nephroma_foliolatum,1

Nephroma_helveticum,1

Nephroma_hensseniae,1

Nephroma_laevigatum,1

Nephroma_occultum,1

Nephroma_parile,1

Nephroma_plumbeum,1

Nephroma_resupinatum,1

Nephroma_skottsbergii,1

Nephroma_sulcatum,1

Nephroma_tangeriense,1

Nephroma_tropicum,1

Nephroma_venosum,1

Anzina_carneonivea,0

Puttea_margaritella,0

Ochrolechia_androgyna,0

Ochrolechia_parella,0

Ochrolechia_peruensis,0

Ochrolechia_subpallescens,0

Ochrolechia_upsaliensis,0

Ochrolechia_yasudae,0

Pertusaria_amara,0

Pertusaria_corallina,0

Pertusaria_dactylina,0

Pertusaria_hemisphaerica,0

Pertusaria_lactea,0

Pertusaria_scaberula,0

Pertusaria_subventosa,0

Varicellaria_culbersonii,0

Varicellaria_rhodocarpa,0

Varicellaria_velata,0

Odontotrema_phacidiellum,0

Odontotrema_phacidioides,0

Degelia_plumbea,1

Erioderma_verruculosum,1

Fuscopannaria_ignobilis,1

Pannaria_rubiginosa,1

Parmeliella_triptophylla,1

Staurolemma_omphalarioides,1

Alectoria_ochroleuca,1

Alectoria_sarmentosa,1

Allocetraria_flavonigrescens,1

Austroparmelina_macrospora,1

Austroparmelina_pruinata,1

Brodoa_intestiniformis,1

Bryocaulon_divergens,1

Bryoria_americana,1

Bryoria_capillaris,1

Bryoria_fremontii,1

Bryoria_furcellata,1

Bryoria_fuscescens,1

Bryoria_glabra,1

Bryoria_implexa,1

Bryoria_nadvornikiana,1

Bryoria_simplicior,1

Bryoria_smithii,1

Bryoria_trichodes,1

Cetraria_islandica,1

Cetraria_kamtczatica,1

Cetraria_nigricans,1

Cetraria_sepincola,1

Cetrariella_commixta,1

Cetrariella_delisei,1

Cetrariella_fastigiata,1

Dactylina_arctica,1

Emodomelanelia_masonii,1

Evernia_prunastri,1

Flavocetraria_cucullata,1

Flavocetraria_nivalis,1

Flavoparmelia_caperata,1

Flavoparmelia_soredians,1

Flavopunctelia_flaventior,1

Hypogymnia_physodes,1

Hypotrachyna_caraccensis,1

Hypotrachyna_degelii,1

Imshaugia_aleurites,1

Letharia_columbiana,1

Masonhalea_richardsonii,1

Melanelia_hepatizon,1

Melanelixia_albertana,1

Melanelixia_californica,1

Melanelixia_fuliginosa,1

Melanelixia_glabratuloides,1

Melanelixia_glabroides,1

Melanelixia_subaurifera,1

Melanelixia_subglabra,1

Melanelixia_villosella,1

Melanohalea_elegantula,1

Melanohalea_exasperata,1

Melanohalea_exasperatula,1

Melanohalea_gomukhensis,1

Melanohalea_halei,1

Melanohalea_infumata,1

Melanohalea_laciniatula,1

Melanohalea_multispora,1

Melanohalea_olivacea,1

Melanohalea_poeltii,1

Melanohalea_septentrionalis,1

Melanohalea_subelegantula,1

Melanohalea_subolivacea,1

Melanohalea_trabeculata,1

Menegazzia_terebrata,1

Montanelia_disjuncta,1

Montanelia_panniformis,1

Montanelia_sorediata,1

Montanelia_tominii,1

Myelochroa_aurulenta,1

Myelochroa_irrugans,1

Nephromopsis_leucostigma,1

Parmelia_saxatilis,1

Parmelia_serrana,1

Parmelina_tiliacea,1

Parmeliopsis_ambigua,1

Parmeliopsis_hyperopta,1

Parmotrema_austrosinense,1

Parmotrema_reticulatum,1

Parmotrema_subtinctorium,1

Parmotrema_tinctorum,1

Platismatia_glauca,1

Pleurosticta_acetabulum,1

Protoparmelia_badia,0

Protousnea_magellanica,1

Pseudephebe_pubescens,1

Pseudevernia_consocians,1

Pseudevernia_furfuracea,1

Punctelia_rudecta,1

Relicina_sydneyensis,1

Tuckermannopsis_chlorophylla,1

Tuckermannopsis_ciliaris,1

Usnea_antarctica,1

Usnea_ceratina,1

Usnea_cornuta,1

Usnea_florida,1

Usnea_glabrescens,1

Usnea_lapponica,1

Usnea_silesiaca,1

Usnea_sphacelata,1

Usnea_strigosa,1

Usnea_subfloridana,1

Usnea_wasmuthii,1

Usnocetraria_oakesiana,1

Vulpicida_canadensis,1

Vulpicida_juniperinus,1

Vulpicida_pinastri,1

Vulpicida_tubulosus,1

Vulpicida_viridis,1

Xanthoparmelia_chlorochroa,1

Xanthoparmelia_conspersa,1

Xanthoparmelia_lipochlorochroa,1

Xanthoparmelia_mougeotii,1

Xanthoparmelia_saxeti,1

Xanthoparmelia_tinctina,1

Xanthoparmelia_wyomingica,1

Peltigera_aphthosa,1

Peltigera_canina,1

Peltigera_degenii,1

Peltigera_horizontalis,1

Peltigera_leucophlebia,1

Peltigera_membranacea,1

Peltigera_praetextata,1

Solorina_crocea,1

Solorina_saccata,1

Agyrium_rufum,0

Loxosporopsis_corallifera,0

Pertusaria_gibberosa,0

Pertusaria_hermaka,0

Pertusaria_leioplaca,0

Pertusaria_paramerae,0

Pertusaria_pertusa,0

Pertusaria_pustulata,0

Phlyctis_agelaea,0

Phlyctis_argena,0

Anaptychia_palmatula,1

Heterodermia_vulgaris,1

Phaeophyscia_orbicularis,1

Physcia_aipolia,1

Physcia_dubia,1

Physconia_muscigena,1

Rinodina_tephraspis,0

Byssoloma_leucoblepharum,0

Byssoloma_subdiscordans,0

Calopadia_foliicola,0

Fellhanera_bouteillei,0

Micarea_alabastrites,0

Micarea_denigrata,0

Micarea_sylvicola,0

Nelsenium_usnicum,0

Placynthium_flabellosum,1

Placynthium_nigrum,1

Placynthium_pannariellum,1

Placynthium_tantaleum,1

Porina_aenea,0

Porina_byssophila,0

Porina_epiphylla,0

Porina_lectissima,0

Porina_nitidula,0

Protothelenella_corrosa,0

Protothelenella_sphinctrinoidella,0

Psilolechia_leprosa,0

Psilolechia_lucida,0

Protoblastenia_calva,0

Psora_decipiens,1

Psora_rubiformis,1

Mycobilimbia_tetramera,0

Byssolecania_variabilis,1

Micarea_adnata,0

Bacidia_rosella,0

Bacidia_schweinitzii,1

Bacidina_arnoldiana,1

Biatora_subduplex,0

Bilimbia_sabuletorum,0

Crocynia_pyxinoides,1

Herteliana_taylorii,0

Lecania_atrynoides,0

Lecania_cyrtella,0

Lopezaria_versicolor,0

Niebla_cephalota,1

Ramalina_complanata,1

Ramalina_farinacea,1

Ramalina_fastigiata,1

Toninia_cinereovirens,1

Catolechia_wahlenbergii,0

Rhizocarpon_disporum,0

Rhizocarpon_geminatum,0

Rhizocarpon_geographicum,0

Rhizocarpon_hochstetteri,0

Rhizocarpon_oederi,0

Rhizocarpon_sphaerosporum,0

Rhizocarpon_superficiale,0

Rhexophiale_rhexoblephara,0

Sagiolechia_protuberans,0

Loxospora_cismonica,0

Loxospora_elatina,0

Loxospora_lecanoriformis,0

Loxospora_ochrophaea,0

Schaereria_corticola,0

Schaereria_dolodes,0

Schaereria_fuscocinerea,0

Scoliciosporum_intrusum,0

Scoliciosporum_umbrinum,0

Calycidium_cuneatum,1

Neophyllis_melacarpa,1

Sphaerophorus_fragilis,1

Sphaerophorus_globosus,1

Sporastatia_polyspora,1

Sporastatia_testudinea,1

Toensbergia_leucococca,0

Lepraria_bergensis,0

Lepraria_incana,0

Lepraria_lobificans,0

Squamarina_cartilaginea,1

Squamarina_gypsacea,1

Squamarina_lentigera,1

Stereocaulon_paschale,1

Stereocaulon_pileatum,1

Stereocaulon_tomentosum,1

Absconditella_lignicola,0

Absconditella_sphagnorum,0

Acarosporina_microspora,1

Cryptodiscus_gloeocapsa,0

Cyanodermella_viridula,1

Schizoxylon_albescens,0

Stictis_populorum,0

Caloplaca_atroflava,1

Caloplaca_chilensis,1

Caloplaca_chlorina,1

Caloplaca_cinnamomea,1

Caloplaca_conversa,1

Caloplaca_granulosa,1

Caloplaca_isidiigera,1

Caloplaca_polycarpa,1

Caloplaca_teicholyta,1

Caloplaca_variabilis,1

Gyalolechia_fulgens,0

Gyalolechia_stipitata,0

Niorma_chrysophthalma,1

Polycauliona_impolita,1

Pyrenodesmia_chalybaea,0

Rufoplaca_scotoplaca,0

Seirophora_californica,1

Seirophora_lacunosa,1

Sirenophila_eos,1

Solitaria_chrysophthalma,1

Stellarangia_elegantissima,1

Teloschistes_exilis,1

Teloschistes_flavicans,1

Teloschistes_hosseusianus,1

Usnochroma_carphinea,1

Usnochroma_scoriophila,1

Variospora_thallincola,1

Variospora_velana,1

Wetmoreana_decipioides,1

Xanthomendoza_fallax,1

Xanthomendoza_oregana,1

Xanthomendoza_poeltii,1

Xanthomendoza_trachyphylla,1

Xanthomendoza_weberi,1

Xanthopeltis_rupicola,1

Xanthoria_aureola,1

Xanthoria_calcicola,1

Xanthoria_parietina,1

Calvitimela_armeniaca,0

Lecidea_aglaea,0

Mycoblastus_affinis,0

Mycoblastus_sanguinarius,0

Tephromela_atra,0

Tephromela_grumosa,0

Violella_fucata,0

Chromatochlamys_muscorum,0

Thelenella_antarctica,0

Thrombium_epigaeum,0

Placopsis_cribellans,1

Placopsis_perrugosa,1

Placynthiella_icmalea,1

Trapelia_coarctata,0

Trapeliopsis_granulosa,0

Umbilicaria_hyperborea,1

Vahliella_californica,1

Vahliella_leucophaea,1

Vahliella_saubinetii,1

Lithographa_tesserata,0

Xylographa_trunciseda,0

Xylographa_vitiligo,0
